# Biologic excipients: Importance of clinical awareness of inactive ingredients

**DOI:** 10.1371/journal.pone.0235076

**Published:** 2020-06-25

**Authors:** Yelena Ionova, Leslie Wilson

**Affiliations:** 1 Department of Bioengineering and Therapeutic Sciences, University of California, San Francisco, California, United States of America; 2 Departments of Medicine and Pharmacy, University of California, San Francisco, California, United States of America; University of Colorado Anschutz Medical Campus, UNITED STATES

## Abstract

Due to the complexity and fragility of biological drug products, several challenges exist in their formulation development. Excipients are added to increase product stability, maintain tonicity, and facilitate drug delivery. The potential implications of these additive substances merit clinical consideration. We assessed the safety risk of excipients on the basis of their type and variability through an assessment framework, which quantifies excipient complexity in 230 biological formulations, and identifies excipient-related adverse events through published case reports. A biologic on average contained 4.45 excipients, half of that found in oral medications. The frequency distribution was heavily skewed towards the most commonly occurring excipients: water (40.4%), sodium chloride (38.3%), polysorbate 80 (28.7%), sucrose (24.4%), and mannitol (20.9%), with 44.4% of formulations not listing the concentration of the most commonly occurring inactive ingredients. A literature search revealed only 17 case reports of excipient-related adverse events, suggesting the need for more clarity for clinicians on the safety of chemical additives. These cases included injection site reactions, anaphylaxis, hyperglycemia, and acute renal failure. With the expansion of the biopharmaceutical market, it is important to consider the safety data of biologic excipients, so that therapy can be tailored appropriately for a specific patient.

## Introduction

Pharmaceutical formulation of a drug product consists of the active pharmaceutical ingredient (API) and excipients—the inactive ingredients that the U.S. Food and Drug Administration (FDA) broadly defines as “any component of a drug product other than an active ingredient” [1]. The API of a biologic is most commonly a growth factor, hormone, interferon, monoclonal antibody, or other peptide or protein. These large molecule compounds can range anywhere from 600 to 150,000 Daltons [2] and most require parenteral administration due to their high molecular weight and low stability properties leading to the risk of denaturalization and proteolytic enzymatic degradation in the gastrointestinal tract.

Excipients are added to the API to increase their stability and preservation, maintain tonicity, and facilitate drug delivery, ensuring the development of the most efficacious medicine that avoids immunogenic or other side effects. Due to the complexity and fragility of these active compounds, several challenges exist in formulation development of a biologic. First, stability and preservation present a significant challenge as the API of a biologic is more unstable than in small molecule drugs. In addition, protein-based therapeutics have a potential to cause an immunogenic response leading to adverse events that are often not discovered until after the medicine is on the market. Lastly, most of these medicines must be developed in a liquid form for compatibility with subcutaneous, intramuscular, or intravenous administration.

Despite the challenges in formulation development, biologics are the fastest growing therapeutic class of medications. In the United States, biologics comprise 40 percent of total spending on prescription drugs [3]. They represented 70 percent of the growth in drug spending from 2010 to 2015 and are forecasted to be the fastest growing sector of the pharmaceutical industry [3]. This includes the development of biosimilar formulations, which are expanding rapidly due to the anticipated patent expirations of many biologics. The U.S. FDA defines a biosimilar as a “biological product that is highly similar to the reference product notwithstanding minor differences in clinically inactive components and that has no clinically meaningful differences in terms of safety, purity or potency from an existing FDA-approved reference product” [4].

As more biologics are developed and adoption of biosimilars spreads, ensuring efficacy, safety and quality of these medicines is an increasing priority. In 2018, U.S. FDA issued the Biosimilars Action Plan (BAP) to stimulate development of biosimilars thereby increasing competition in the biologics marketplace. Part of this plan focused on development of scientific tools and resources that would enhance understanding of appropriate analytical methods to demonstrate biosimilarity and thus efficacy of the medicine relative to the reference product [4]. A large part of ensuring safety and quality of medicines involves appropriate excipient selection considering for instance that 92.8% of oral medicines contain at least one potential allergen in its formulation [5]. Recent research in oral medications has shown that the “inactive” ingredients are not as inert as the name suggests [5]. Similarly, inactive ingredients in parenterally administered medicines have been associated with increased sensation of pain at the injection site, and a review of factors impacting immunogenicity of biologics has identified a number of inactive ingredients as potential causes [6–7]. These ingredients can exert adverse effects on individuals with known sensitivities and intolerances, especially on vulnerable pediatric and elderly populations with serious and life-threatening diseases that might require treatment with unstable biological medicines.

Our objective is to assess the potential safety risk of biological formulations by correlating with the type and variability of excipients used in the product development. We aim to propose an excipient assessment framework to quantify the complexity of biological products by excipient use frequency and their concentrations, and to identify the most commonly occurring excipients found in large molecule medicines with reported adverse events based on published case reports. We argue that excipients play a critical role in the safety profile of a biologic and their potential adverse effects warrant serious clinical consideration to ensure safe medicine for each patient.

## Methods

Our methods included a classification of biologic excipients, identification of their complexity to identify their relevance, and a broad literature search to identify the extent of specifically documented excipient adverse effects.

### Classification

Excipients used in biological medicines were categorized by function, class, and type of product by adapting a variety of existing sources (Table 1). Further expansion and formal adoption of this classification system to better fit biologics is warranted.

**Table 1:** Excipient functional category, class and types used in biologics.

Functional Category^a^	Excipient Class^b^	Types
pH Modifier (Acidifying/Alkalizing/ Buffering Agent)	Buffering Agents	Acetate, Citrate, Tartrate, Histidine, Glutamate, Phosphate, Tris, Glycine, Bicarbonate, Succinate, Sulfate, Nitrate
Tonicity Agent	Tonicity Modifiers	Mannitol, Sorbitol, Lactose, Dextrose, Trehalose, Sodium Chloride, Potassium Chloride, Glycerol, Glycerin
Bulking Agent	Sugars and polyols	Sucrose, Trehalose, Glucose, Lactose, Sorbitol, Mannitol, Glycerol
	Amino Acids	Arginine, Aspartic Acid, Glutamic acid, Lysine, Proline, Glycine, Histidine, Methionine, Alanine,
	Polymers and proteins	Gelatin, PVP, PLGA, PEG, dextran, cyclodextrin and derivatives, starch derivatives, HSA, BSA
Wetting and/or Solubilizing Agent	Surfactants	Polysorbate 20 (Tween 20), Polysorbate 80 (Tween 80), Poloxamer (Pluronic F68 and F127), Triton X-100, Brij 30, Brij 35
Antioxidant	Antioxidant Preservatives	Histamine, methionine, ascorbic acid, glutathione, vitamin E, poly(ethylenimine)
Antimicrobial Preservative	Antimicrobial Preservatives	Benzyl alcohol, metacresol, phenol, 2-phenoxyethanol
Chelating and/or Complexing Agents	Chelator Preservatives	Edetate disodium, diethylenetriamine pentaacetic acid (DTPA), citric acid, hexaphosphate, thioglycolic acid, zinc

^a^Functional category modified from USP-NF 42–37 [8].

^b^Excipient class adapted from “Excipient selection in biologics and vaccines formulation development” [9] and “Excipients Used in Biotechnology Products” [10].

### Complexity

**Fig 1:**
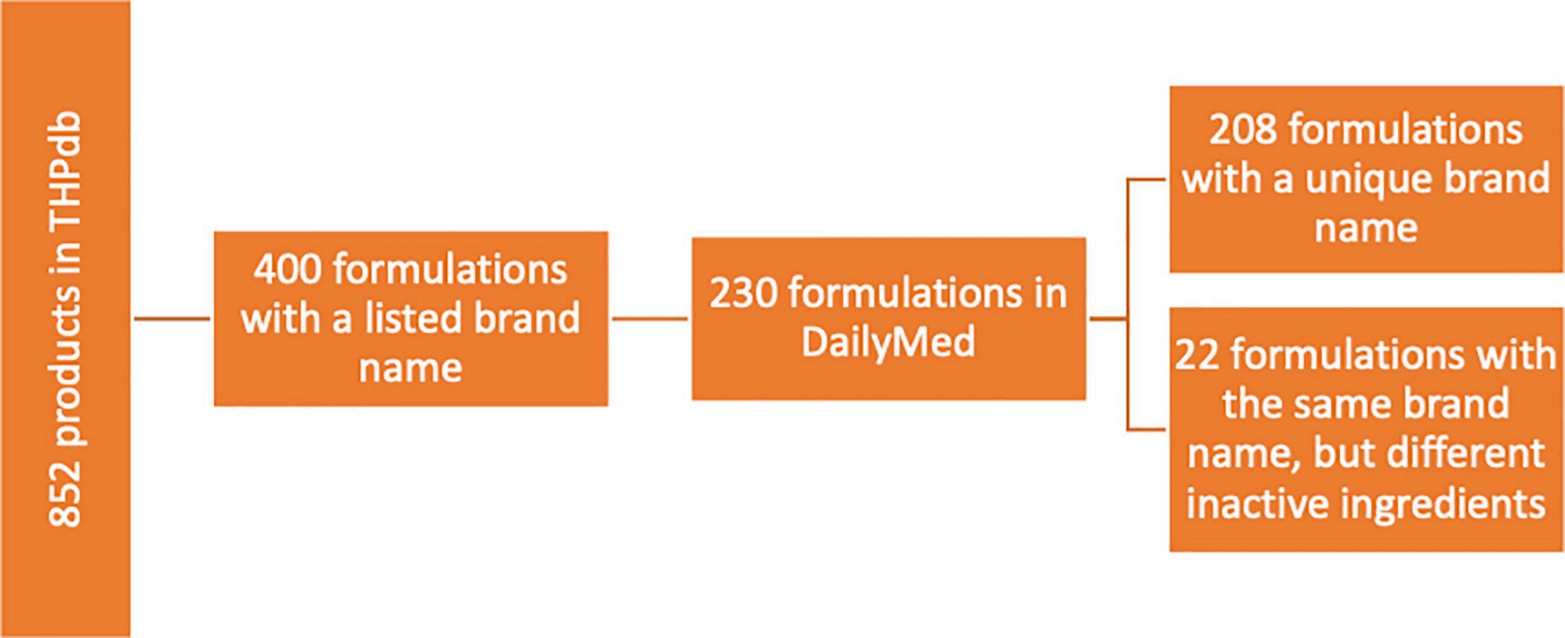
Data extraction methodology.

We describe the complexity as consisting of the variability in excipient selected for use across formulations and their concentrations in weight by volume. Complexity information about currently marketed biological medicines and their formulations was extracted from two primary databases. The therapeutic peptides database (THPdb) is a comprehensive database of approved therapeutic peptides and proteins that provides information about their indication, pharmacokinetic and pharmacodynamic properties, formulation and other factors [2]. DailyMed is an official provider of U.S. FDA labeling information (package inserts) that includes a comprehensive list of inactive ingredients in an approved medication and also the amount of active and inactive ingredients in each formulation [11]. The DailyMed search was conducted using the brand name of the medication listed in THPdb. A few brand names contained multiple labels, each listing different excipients, in which case each preparation was considered as its own unique formulation (Fig 1). We calculated the Gini coefficient to measure the variation in use frequency of inactive ingredients [4]. A Gini coefficient is a measure of disparity that ranges from 0 (perfect equality) to 1 (perfect inequality), and is often used to determine economic equality. It is calculated as a ratio of the area above the distribution line, but below the line of perfect equality, and the entire area below the line of perfect equality (S1 Fig). Applying this measure to frequency of excipients use, a Gini index of 0 indicates that the use of an inactive ingredient is equal to the use of every other inactive ingredient; in other words each inactive ingredient is used at the same rate. As the Gini coefficient moves towards 1, the rate of excipient use shifts towards more commonly occurring inactive ingredients. A Gini index of 1 indicates that only one ingredient is present across all medicines and no other inactive ingredients are used [4].

The concentration of each inactive ingredient was recorded if listed under the “Ingredients and Appearance” section of the DailyMed label. From these records, the average concentration of inactive ingredients was calculated for medications with multiple volumes and for formulations with varying concentrations of inactive ingredients. In addition, we calculated the range and average concentration for the most commonly occurring inactive ingredients (excipients occurring in more than 10 formulations). The concentrations of inactive ingredients were recorded in weight by volume. Formulation labels that did not list concentrations of inactive ingredients (N = 318 excipients) and formulations with unspecified volume (N = 15 excipients) were excluded from this analysis (Fig 1).

### Adverse effect profiles

A literature search was conducted to understand the extent of adverse effects of excipients in biologics. We narrowed the focus of our search to the largest three classes of excipients: surfactants, sugars and polyols, and preservatives. More specific adverse effect profiles of excipients in these three categories were constructed based on a PubMed search using three different strategies. First, we looked at adverse event cases after administration of a biologic formulation with high concentrations of a particular excipient. For this portion of the review, we narrowed our search to the surfactants, sugars and polyols, and preservatives that occurred in more than 10 formulations. The excipients that were examined in this section were polysorbate 80, sucrose, mannitol, polysorbate 20, citric acid, metacresol, sorbitol, phenol, and zinc. The search terms used were excipient name and generic or brand name of the drug containing that excipient. The top 10 percent of formulations containing the highest concentrations of the excipient were included in the search (Table 2).

**Table 2:** Number of search results of most commonly occurring excipients with highest concentrations.

Excipient	Number of Formulations Included in the Search	Number of Case Reports
**Polysorbate 80**	6	1
**Sucrose**	7	1
**Mannitol**	5	0
**Polysorbate 20**	3	2
**Citric acid**	2	0
**Metacresol**	2	1
**Sorbitol**	2	0
**Phenol**	2	0
**Zinc**	1	1

Our second approach focused on a search of all adverse drug reactions to excipients in biologics followed by a third and narrower search focusing specifically on the anaphylaxis and hypersensitivity case reports due to a surfactant, sugar or polyol, or preservative (Table 3). The reviewed surfactants were polysorbate 80, polysorbate 20, and poloxamer 188. The sugars and polyols group included sucrose, mannitol, sorbitol, trehalose, lactose, and glycerol or glycerin. Finally, the preservatives that were included were citric acid, metacresol, phenol, zinc, methionine, glutathione, benzyl alcohol, and edetate disodium. The terms used in the first phase of the search were either the excipient class or specific excipient names, “adverse drug reaction,” “adverse drug effect,” or “adverse effect,” and the terms “biologic,” “biosimilar,” “monoclonal antibody,” “immunoglobulin,” “biopharmaceuticals,” or “biotherapeutic.” The second phase of the search included the following terms: excipient class or excipient names, “anaphylaxis,” “hypersensitivity” or “allergic reaction,” and again the terms “biologic,” “biosimilar,” “monoclonal antibody,” “immunoglobulin,” “biopharmaceuticals,” or “biotherapeutic.” The filters for “humans” species and “case reports” article types were applied to all literature searches in order to isolate human case studies only.

**Table 3:** Number of search results of adverse drug reactions (ADRs) and anaphylaxis and hypersensitivity cases due to an excipient in a biologic formulation.

	Number of Search Results	
Excipient Class and Name	Adverse Drug Reaction or Adverse Effect	Anaphylaxis or Allergic Reaction
**Surfactant or Polysorbate**	3	6
**By Excipient Name** polysorbate 80, polysorbate 20, poloxamer 188	1	2
**Sugar or Polyol**	4	4
**By Excipient Name** sucrose, mannitol, sorbitol, trehalose, lactose, glycerol or glycerin	9	17
**Preservative**	0	0
**By Excipient Name** citric acid, metacresol, phenol, zinc, methionine, glutathione, benzyl alcohol, edetate disodium	11	17

## Results

### Biologics and their inactive ingredients

A total of 230 formulations are included in this analysis encompassing 208 unique drugs (S1 Table). Of these, 188 (90.4%) are designed for intramuscular, intravenous, or subcutaneous administration. Six medicines (2.9%) are taken by oral route, followed by 4 topical and intravitreal drugs (1.9%) in each respective drug delivery category. Almost a quarter of the medicines (50 biologics) are monoclonal antibodies. The final list of 230 formulations contained a total of 1,024 inactive ingredients, of which 138 were unique. A few ingredients that differed only in their hydrous state were combined into one unique entry resulting in 120 unique inactive ingredients (S2 Table). Sodium chloride, polysorbate 80, sucrose, and mannitol are the most common excipients after water. Polysorbate 20 and metacresol are 9^th^ and 19^th^ most common ingredients, respectively, out of 120 different excipients.

### Complexity of formulations

**Fig 2:**
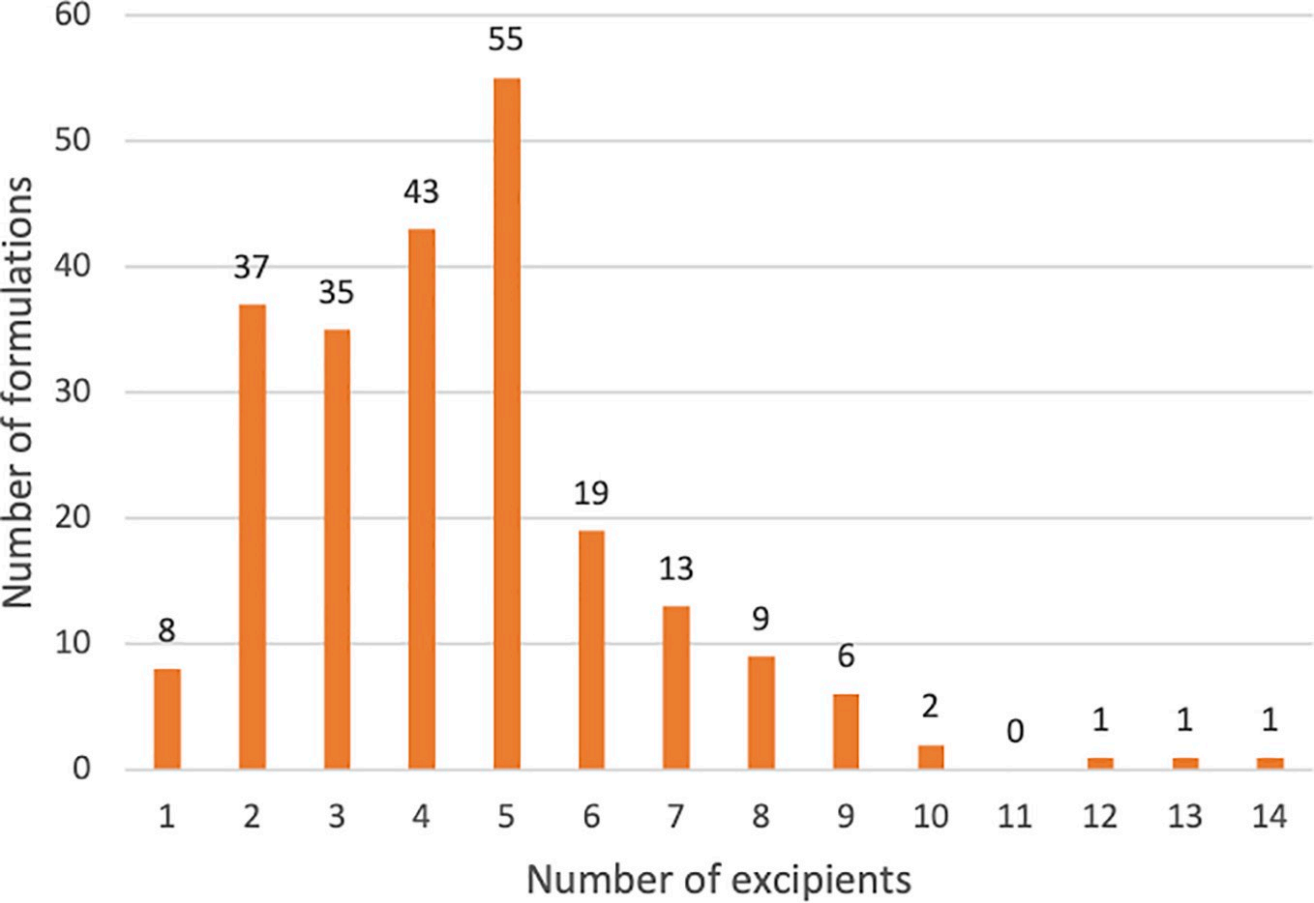
Distribution of inactive ingredients in biologic formulations.

**Fig 3:**
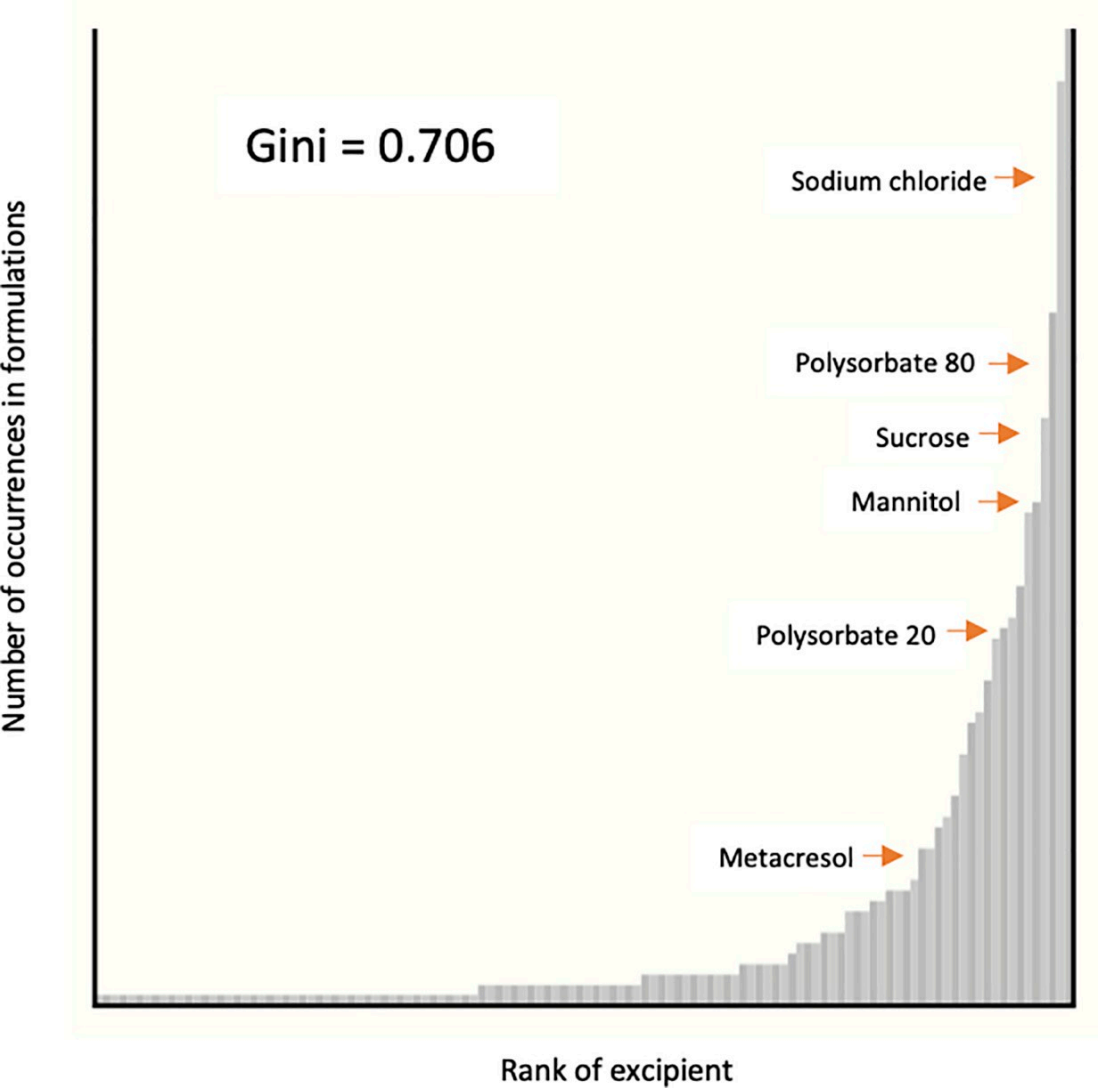
Frequency of inactive ingredients in biologic formulations expressed as a Gini coefficient.

Complexity of biological formulations is defined by the frequency of occurrence of inactive ingredients and their concentration. A biological drug product on average contained 4.45 excipients, half of that found in small molecule oral medications that contain 8.8 excipients on average [4]. The number of inactive ingredients in the 230 analyzed formulations ranged from 1 to 14 (median = 4) with only five biological products containing 10 or more excipients (Fig 2). The most common inactive ingredients are water (40.4%), sodium chloride (38.3%), polysorbate 80 (28.7%), and sucrose (24.3%), occurring in over 50 formulations. Another twenty different excipients are found in 11 to 50 biological formulations. A little over 80 percent of all excipients found in biologics occur in 10 or fewer formulations, with half of those only occurring once. We calculated a Gini coefficient of 0.706 which indicates that the frequency distribution of excipients is skewed heavily towards fewer inactive ingredients, which are the most commonly occurring ones (Fig 3). A complete list of inactive ingredients that occur in biologics is listed in S3 Table.

On average 44.4% of the biological formulations do not list the concentration of the most commonly occurring inactive ingredients. Metacresol and zinc were the only commonly occurring ingredients with listed amounts in over 90 percent of formulations followed by phenol (72.7%), mannitol (70.8%), sodium phosphate dibasic (68.1%) and polysorbate 20 (66.7%) (Table 4). Sugars and polyols occur at the highest concentrations followed by arginine hydrochloride and glycine amino acids. The average concentration of sucrose in 34 formulations is 72.7 mg per 1 ml. Sorbitol and mannitol occur at average concentrations of 42.71 mg/ml and 30.62 mg/ml, respectively. In comparison, the average concentrations of polysorbate 80 and polysorbate 20 are 0.38 mg/ml and 0.14 mg/ml, respectively (Table 4).

**Table 4:** Concentrations of commonly occurring inactive ingredients in biologics.

Excipient	Number of formulations containing the excipient	Percentage of formulations with listed concentration of the excipient	Number of formulations included in mass analysis	Minimum mass (mg) per 1 ml of volume	Maximum mass (mg) per 1 ml of volume	Average mass (mg) per 1 ml of volume
**water**	93	0	0	--	--	--
**sodium chloride**	88	52.3% (46)	45	0.01	11.69	5.73
**polysorbate 80**	66	57.6% (38)	37	0.01	5.00^a^	0.38^a^
**sucrose**	56	60.7% (34)	31	4.00	500.00	72.20
**mannitol**	48	70.8% (34)	33	1.60	54.50	30.62
**sodium phosphate dibasic**	47	68.1% (32)	31	0.20	13.45	2.25
**histidine**	40	62.5% (25)	23	0.04	7.76	2.03
**sodium hydroxide**	37	0	0	--	--	--
**polysorbate 20**	36	66.7% (24)	23	0.01	0.40	0.14
**sodium phosphate monobasic**	35	65.7% (23)	21	0.22	12.70	2.12
**hydrochloric acid**	31	3.2% (1)	0	--	--	--
**sodium citrate**	28	50.0% (14)	13	0.30	12.94	4.56
**glycine**	27	37.0% (10)	10	0.11	22.50	12.46
**albumin (human)**	24	50.0% (12)	12	1.00	50.00	11.61
**citric acid**	20	60.0% (12)	11	0.06	5.25	1.13
**sodium acetate**	18	50.0% (9)	9	0.10	6.80	1.94
**histidine monohydrochloride**	17	58.8% (10)	10	0.48	4.28	1.76
**acetic acid**	15	40.0% (6)	6	0.41	2.25	0.96
**metacresol**	15	93.3% (14)	14	1.50	3.15	2.32
**sorbitol**	12	58.3% (7)	7	20.00	50.00	42.71
**arginine hydrochloride**	11	54.5% (6)	6	5.37	42.10	20.82
**phenol**	11	72.7% (8)	8	0.65	5.50	2.87
**zinc**	11	90.9% (10)	10	0.00	0.07	0.03

List of commonly occurring ingredients in biologics, how often the concentration of excipients are reported, and concentration range and average of each ingredient.

^a^Reteplase drug label records the concentration of polysorbate 80 at 5 mg in 1 ml. The second highest concentration drops down to 1.04 mg in 1 ml significantly lowering the average from 0.38 mg/ml to 0.25 mg/ml.

### Adverse reactions associated with excipients in biologics

Our review of surfactants, sugars and polyols, and preservatives produced only 17 case reports of adverse events [15–28]. These cases included injection site reactions, multiple reports of anaphylaxis, hyperglycemia, and acute renal failure.

Of the 17 case reports found, six were due to an adverse effect to a surfactant [12–17]. These include an 80-year-old female patient who developed an injection site reaction due to polysorbates in PCSK9i formulations [14]. Another was a 28-year-old woman with plaque psoriasis who developed urticariform symptoms after an injection of adalimumab and subsequently ustekinumab. Both contained polysorbate 80 which was determined to be the cause of her reaction based on a cutaneous skin test [15]. A third skin-prick confirmed excipient-caused adverse event was of a teenager who developed an anaphylactic reaction 10 minutes after an injection of omalizumab that contained polysorbate 20 [16]. Two more patients experienced similar anaphylactoid adverse reactions to polysorbate 20 in omalizumab in a separate case report [17]. Anti-asthmatic monoclonal antibody agent omalizumab contains the highest concentration of polysorbate 20 at 0.36 mg/ml in a vial and 0.4 mg/ml in a pre-filled syringe formulation.

A literature search resulted in 23 case reports of adverse effects due to sugars and polyols; however, 10 were excluded because they were not caused by the excipients with three case reports confirmed through testing that the adverse effect was not due to any inactive ingredient in the formulation. Nevertheless, there were 10 cases of adverse effects due to sugars and polyols found [18–27]. Of these, four cases were due to sucrose-induced reactions; four cases were due to mannitol; one report implicated alpha-gal as the possible mechanism for anaphylaxis after a zoster vaccine [18]; and finally, one report described the anaphylactic reaction experienced by a 6-year-old boy with a history of milk-allergy after inhalation of Inavir containing lactose [19]. From the adverse effects due to sucrose, three case reports documented patients that experienced acute renal failure attributed to significant amounts of sucrose in the intravenous immunoglobulin (IVIg) formulations [20–22]. The other case report described two patients with type 2 diabetes treated with omalizumab for severe persistent asthma that developed hyperglycemia implicating the high concentration of the sucrose excipient in omalizumab as the cause [23]. Another report was a phase I study of mannitol used to disrupt restrictions from the blood-brain-barrier followed by cetuximab administration for recurrent malignant glioma. Even though direct association was not addressed, the study reported that 2 patients experienced tolerable rash and 1 patient experienced anaphylaxis [24]. Three other cases of mannitol-induced adverse effects described anaptyctic reactions, one of which resulted in intraoperative death due to mannitol in thymoglobulin [25–27].

In examining literature for adverse effects to preservatives in biological products, one isolated case report revealed zinc excipient as the offending agent causing generalized allergy in an 11-year-old with type I diabetes treated with an insulin pump [28].

## Discussion

In order to expand the knowledge of the complexity of biologic formulations, as defined in the Methods section, and improve understanding of the extent of reported adverse events due to the inactive ingredients in biologics, we first performed databases analyses quantifying the frequency of excipient occurrence and their concentrations, and then a literature search to identify case studies of excipient-related adverse events. We found high variability in excipient selection and concentration, and identified several case reports of adverse events to a variety of classes of excipients in biologic formulations.

Although the average number of excipients utilized in biologic formulations was found to be half of those added to small molecule medicines, the distribution of 120 unique excipients among 230 medicines ranging between 1 and 14 ingredients per formulation indicates high variability. Applying the Gini coefficient as a metric for analyzing frequency of distribution, we showed that the distribution is skewed towards the most commonly occurring ingredients. The Gini coefficient is a well-known economic measure used to quantify income inequalities by looking at the distribution of income in a population. However, it has been used to describe the variation in other contexts including a distribution of excipients among oral medicines [4, 29]. Our findings also confirmed that the concentrations of inactive ingredients in biologics is largely not reported by the manufacturers. Knowing the concentration of the inactive ingredient in a formulation may help to uncover its potential role in causing an adverse effect although even small amounts of allergens in a medication may induce serious anaphylactic reactions [4]. Variability in biologic formulations on the basis of excipient selection and use may be clinically important to identify groups of patients that may be more susceptible to certain adverse effects.

Polysorbate 20 and polysorbate 80 are the most common surfactants occurring in biologics. Surfactants are widely used in the production process as raw material in the purification, filtration, transportation, lyophilization, and storage, and are added to the final solution to stabilize proteins, prevent aggregation, and assist in protein folding. Despite having a stabilizing effect on API, polysorbates are subject to oxidative degradation resulting in formation of residual peroxides and other reactive oxidative species. The increase in these degradation products has been reported in an interleukin-2 formulation containing polysorbate 80 [30]. Polysorbates also act as photoenhancers, which may lead to photooxidation. Studies have shown that the photostability of antibodies is significantly affected by the quality and type of the surfactant in the formulation [31, 32].

Considering the chemical instability of these ingredients, the high prevalence of use, and their varying concentrations, clinicians should be informed about the potential adverse events in some patients for biologics containing these excipients. Although data on the clinical effects of these compounds is limited, we have enough examples to support the importance of increasing clinical awareness on the potential of some excipients to have serious consequences in certain patients. For instance, a literature search identified three patients that experienced anaphylactoid reaction due to polysorbate 20 in a monoclonal antibody omalizumab. According to U.S. FDA Adverse Event Reporting System (AERS), since its approval in 2003 through 2018, there were a total of 1,718 reports of anaphylactic reaction and 1,604 reports of hypersensitivity to omalizumab [33]. Although U.S. FDA does not classify the adverse event reports on the basis of excipients, the large number of reported anaphylactoid events and hypersensitivity reactions to the FDA suggests that the inactive ingredient in omalizumab as a possible cause should be further investigated.

In our analysis, sucrose is the fourth most common excipient found in biologics occurring in 24.4% of the formulations, followed by mannitol (20.9%), sorbitol (5.2%), trehalose (3.9%), and lactose (3.0%). Of note is that the concentration of these compounds ranges widely. For instance, sucrose was added at a concentration of 4 mg/ml to an immunosuppressive monoclonal antibody basiliximab, but another immunosuppressive agent belatacept contains 500 mg/ml of sucrose. Sugars and polyols provide additional stability to protein therapeutics in liquid and lyophilized products. The stabilizing effect is dependent on the concentration of the excipient, causing high amounts to be used in formulations. While limited, the 10 case reports of sugar- and polyol-related adverse events could indicate that this category is associated with serious and even fatal adverse events in patients. Therefore, patients with a history of an allergic reaction or intolerance to polyols and patients with certain disease states such as diabetes and renal insufficiency should be more closely monitored by clinicians if they are receiving a formulation with mannitol, sorbitol or a high concentration of sugar excipient.

In building the adverse effect profiles, we also examined the role and safety of preservatives added to biological formulations. Antioxidants and chelator preservative agents are added to formulations to minimize oxidation reactions and maintain the stability and safety of biologics. Antimicrobial agents are added to prevent microbial contamination especially in multidose formulations that require multiple opening and closure of the container. Metacresol and phenol are believed to be the cause of skin reactions at the infusion sites according to a variety of assays performed on multiple insulin formulations, which showed that exposure to phenolic excipients induces proinflammatory response and cell death, thus stimulating additional inflammatory processes [34]. However, we isolated only one case report of an adverse event to a preservative in an insulin formulation. Knowing that zinc in insulin formulations may induce generalized allergy may help physicians better tailor diabetes management therapy.

With the rapidly expanding biopharmaceutical market, adverse drug reactions to biological products will continue to be a growing concern. These adverse event occurrences are often not described in literature and further analysis to include adverse effects beyond case reports is warranted. Nevertheless, this broad, but focused literature search although not a systematic review supports the notion that while excipients are essential components of the formulation, they could also affect the safety profile of a biologic and may be the cause of certain adverse events in patients.

## Conclusions

Biological drug products contain fewer inactive ingredients than small molecule medicines. Nevertheless, the raw materials that are used in biologics development and added as excipients for stability, preservation, and facilitation of drug delivery play a critical role in the final medicine formulation. The high frequency of occurrence of a number of these inactive ingredients, their varying concentrations, combined with several reported cases of adverse events to these chemical additives suggest that excipients might not be negligible, inert ingredients.

In November 2019, U.S. FDA released a draft document outlining best practices in drug and biological product postmarket safety surveillance [35]. This draft includes processes for detecting adverse effects possibly related to excipients in generic medicines, but similar processes are not addressed for biologics or biosimilars. The FDA and a standards-setting organization such as the United States Pharmacopeial Convention can provide tools and guidance on more comprehensive documentation on the safety of excipients to further our understanding of their clinical risk. In the meantime, clinicians can provide further investigational evidence of adverse reactions to medications through skin-prick testing and other diagnostic measures to confirm or rule out the excipient as the cause. Depending on the severity of a reaction, these patients should be switched to an alternative formulation or monitored more closely during and after administration of the formulation.

Overall, these findings establish a safety assessment framework of biologic formulations that can be utilized to gauge the potential clinical impact of excipients. Recognizing a particular excipient in biologic therapy and its association with a reported adverse event may provide additional evidence for patients’ reactions to a particular medication. With the expansion of the biopharmaceutical market and availability of more data, biologic medication therapy can be tailored appropriately for a specific patient. Aside from clinical implications, recognizing the potential adverse risk of additive ingredients in protein-based therapeutics may drive regulatory initiatives and stimulate more innovative, safer alternative formulations during pharmaceutical development of biological drug products.

## Supporting information

S1 Fig Inequality measurement: The Gini coefficient. (PDF)

S1 Table Biological drug products, therapeutic category, and route of administration. List of all biological drug products considered in this analysis.
(PDF)

S2 Table Inactive ingredients that were combined into a single excipient entry. (PDF)

S3 Table Occurrence frequency of inactive ingredients in biological formulations. List of all ingredients that occur in biological formulations. Percentage occurrence refers to the fraction of all biological formulations analyzed that contain the ingredient.
(PDF)

## References

[1] U.S. Food and Drug Administration. Inactive Ingredient Field Descriptions. Available from: https://www.fda.gov/drugs/informationondrugs/ucm075230.htm. Last updated 15 May 2015.

[2] Usmani SS, Bedi G, Samuel JS, Singh S, Kalra S, Kumar P, et al THPdb: Database of FDA-approved peptide and protein therapeutics. PLoS One 2017; 12(7):e0181748 10.1371/journal.pone.0181748 28759605 PMC5536290

[3] U.S. Food and Drug Administration. Remarks from FDA Commissioner Scott Gottlieb, M.D., as prepared for delivery at the Brookings Institution on the release of the FDA’s Biosimilars Action Plan. July 2018.

[4] U.S. Food and Drug Administration. Biosimilars Action Plan: Balancing Innovation and Competition. July 2018. Available from: https://www.fda.gov/media/114574/download

[5] Reker D, Blum SM, Steiger C, Anger KE, Sommer JM, Fanikos J, et al “Inactive” ingredients in oral medications. Sci Transl Med 2019; 11(483): eaau6753 10.1126/scitranslmed.aau6753 30867323 PMC7122736

[6] Usach I, Martinez R, Festini T, Peris JE. Subcutaneous Injection of Drugs: Literature Review of Factors Influencing Pain Sensation at the Injection Site. Adv Ther 2019; 36:2986–2996. 10.1007/s12325-019-01101-6 31587143 PMC6822791

[7] Singh SK. Impact of Product-Related Factors on Immunogenicity of Biotherapeutics. J Pharm Sci 2011; 100(2): 354–87. 10.1002/jps.22276 20740683

[8] United States Pharmacopeia 42 –National Formulary 37 (USP 42-NF 37). United States Pharmacopeial Convention Inc, 2019.

[9] Medi MB, Chintala R. Excipient selection in biologics and vaccines formulation development. European Pharmaceutical Review [Internet]. 2014 4 11(1). Available from: https://www.europeanpharmaceuticalreview.com/article/24136/excipient-selection-biologics-vaccines-formulation-development/

[10] Chi, EY. Excipients used in biotechnology products. Pharmaceutical Excipients: Properties, Functionality, and Applications in Research and Industry. Available from: https://onlinelibrary.wiley.com/doi/pdf/10.1002/9781118992432.ch4

[11] Labels Drug. DailyMed. U.S. National Library of Medicine. Available from: https://dailymed.nlm.nih.gov/dailymed/10.1080/15360280801989377 28792816

[12] Carbonell A, Escudero AI, Miralles JC, González A, Navarro C, Cardona P, et al Anaphylaxis Due to Poloxamer 238. J Investig Allergol Clin Immunol 2018; 28(6): 419–420. 10.18176/jiaci.0298 30530388

[13] Caballero ML, Lluch-Bernal M, Vilà-Nadal G, Lluncor M, Quirce S. IgE-Mediated Anaphylaxis Induced by Macrogol 6000. J Investig Allergol Clin Immunol 2016; 26(6): 398–400. 10.18176/jiaci.0089 27996956

[14] Singh SK, Mahler HC, Hartman C, Stark C. Are Injection Site Reactions in Monoclonal Antibody Therapies Caused by Polysorbate Excipient Degradants? J Pharm Sci 2018; 107(11): 2735–2741. 10.1016/j.xphs.2018.07.016 30055223

[15] Pérez-Pérez L, García-Gavín J, Piñeiro B, Zulaica A. Biologic-induced urticaria due to polysorbate 80: usefulness of prick test. Brit J Dermatol 2011; 164(5): 1119–20. 21219296 10.1111/j.1365-2133.2011.10220.x

[16] Perino E, Freymond N, Devouassoux G, Nicolas JF, Berard F. Xolair-induced recurrent anaphylaxis through sensitization to the excipient polysorbate. Ann Allergy Asthma Immunol 2018; 120(6):664–666. 10.1016/j.anai.2018.02.018 29481891

[17] Price KS, Hamilton RG. Anaphylactoid reactions in two patients after omalizumab administration after successful long-term therapy. Allergy Asthma Proc 2007; 28(3): 313–9. 10.2500/aap.2007.28.3003 17619560

[18] Stone CA Jr, Hemler JA, Commins SP, Schuyler AJ, Phillips EJ, Peebles RS Jr, et al Anaphylaxis after zoster vaccine: Implicating alpha-gal allergy as a possible mechanism. J Allergy Clin Immunol 2017; 139(5): 1710–1713. 10.1016/j.jaci.2016.10.037 27986511 PMC5420485

[19] Morikawa M, Kanemitsu Y, Tsukamoto H, Morikawa A, Tomioka Y. A case of anaphylaxis in the pediatric patient with milk allergy due to traces of milk protein in the lactose used as an excipient of Inavir inhalation. Arerugi 2016; 65(3): 200–5. 10.15036/arerugi.65.200 27193929

[20] Wajanaponsan N, Cheng SF. Acute renal failure resulting from intravenous immunoglobulin therapy. Hawaii Med J 2004; 63(9): 266–7. 15540524

[21] Subtirelu MM, Flynn JT, Schechner RS, Pullman JM, Feuerstein D, Del Rio M. Acute renal failure in a pediatric kidney allograft recipient treated with intravenous immunoglobulin for parvovirus B19 induced pure red cell aplasia. Pediatr Transplant 2005; 9(6): 801–4. 10.1111/j.1399-3046.2005.00379.x 16269055

[22] Haskin JA, Warner DJ, Blank DU. Acute Renal Failure after Large Doses of Intravenous Immune Globulin. Ann Pharmacother 1999; 33(7–8): 800–3. 10.1345/aph.18305 10466908

[23] Yalcin AD, Gorczynski RM, Cilli A, Strauss L. Omalizumab (anti-IgE) therapy increases blood glucose levels in severe persistent allergic asthma patients with diabetes mellitus: 18 month follow-up. Clin Lab 2014; 60(9): 1561–4. 10.7754/clin.lab.2013.130302 25291953

[24] Chakraborty S, Filippi CG, Wong T, Ray A, Fralin S, Tsiouris AJ. Superselective intraarterial cerebral infusion of cetuximab after osmotic blood/brain barrier disruption for recurrent malignant glioma: phase I study. J Neurooncol 2016; 128(3): 405–15. 10.1007/s11060-016-2099-8 26945581

[25] Findlay SR, Kagey-Sobotka A, Lichtenstein LM. In vitro basophil histamine release induced by mannitol in a patient with a mannitol-induced anaphylactoid reaction. J Allergy Clin Immunol 1984; 73(5 Pt 1): 578:83. 10.1016/0091-6749(84)90514-1 6201521

[26] Hegde VL, Venkatesh YP. Anaphylaxis to excipient mannitol: evidence for an immunoglobulin E-mediated mechanism. Clin Exp Allergy 2004; 34(10): 1602–9. 10.1111/j.1365-2222.2004.02079.x 15479277

[27] Roncati L, Barbolini G, Scacchetti AT, Busani S, Maiorana A. Unexpected death: anaphylactic intraoperative death due to Thymoglobulin carbohydrate excipient. Forensic Sci Int 2013; 228(1–3): e28–32. 10.1016/j.forsciint.2013.02.036 23540837

[28] Gin H, Aubertin J. Generalized allergy due to zinc and protamine in insulin preparation treated with insulin pump. Diabetes Care 1987; 10(6): 789–90. 10.2337/diacare.10.6.789 3322735

[29] Muelas MW, Mughal F, O’Hagan S, Day PJ, Kell DB. The role and robustness of the Gini coefficient as an unbiased tool for the selection of Gini genes for normalizing expression profiling data. Sci Rep 2019; 9(1): 17960 10.1038/s41598-019-54288-7 31784565 PMC6884504

[30] Ha E, Wang W, Wang YJ. Peroxide formation in polysorbate 80 and protein stability. J Pharm Sci 2002; 91(10):2252–64. 10.1002/jps.10216 12226852

[31] Singh SR, Zhang J, O’Dell C, Hsieh MC, Goldstein J, Liu J, et al Effect of Polysorbate 80 Quality on Photostability of a Monoclonal Antibody. AAPS PharmSciTech 2012 13(2): 422–430. 10.1208/s12249-012-9759-6 22362139 PMC3364395

[32] Mahjoubi N, Fazeli A, Dinarvand R, Khoshayand MR, Shekarchi M, Fazeli MR. Effect of Nonionic Surfactants (Dodecyl Maltoside and Polysorbate 20) on Prevention of Aggregation and Conformational Changes of Recombinant Human IFNβ_1b Induced by Light. Iran J Pharm Res 2017; 16(1): 103–111. 28496465 PMC5423237

[33] U.S. Food and Drug Administration. U.S. FDA Adverse Event Reporting System. Available from: https://www.fda.gov/drugs/questions-and-answers-fdas-adverse-event-reporting-system-faers/fda-adverse-event-reporting-system-faers-public-dashboard. Last updated September 2019.

[34] Weber C, Kammerer D, Streit B, Licht A. Phenolic excipients of insulin formulations induce cell death, pro-inflammatory signaling and MCP-1 release. Toxicol Rep 2015; 2: 194–202. 10.1016/j.toxrep.2014.11.019 28962351 PMC5598374

[35] U.S. Food and Drug Administration. Best Practices in Drug and Biological Product Postmarket Safety Surveillance for FDA Staff–Draft. 11 2019 Available from: https://www.fda.gov/media/130216/download 10.18553/jmcp.2019.19041

