# Supplementary figures and images for: Biologic excipients: Importance of clinical awareness of inactive ingredients
Source: PLoS One. 2020 Jun 25;15(6):e0235076. doi: 10.1371/journal.pone.0235076 (PMC7316246; doi:10.1371/journal.pone.0235076)

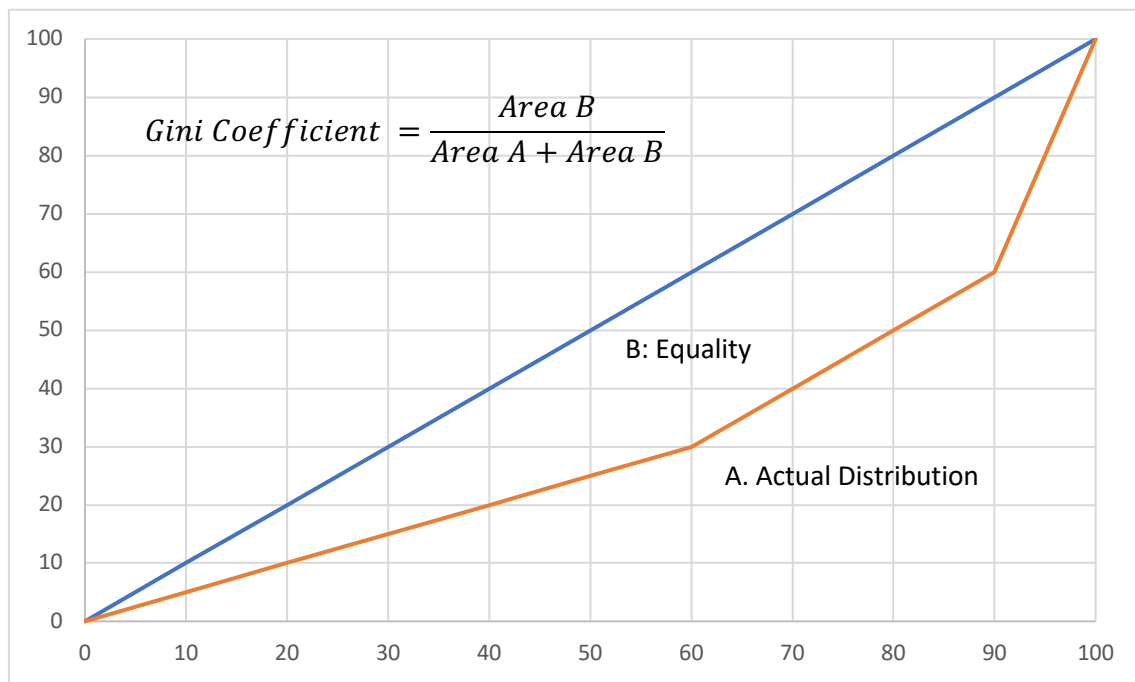

Supplement: S1 Fig — (PDF) [file pone.0235076.s001.pdf]
