# Supplementary material for: Biologic excipients: Importance of clinical awareness of inactive ingredients
Source: PLoS One. 2020 Jun 25;15(6):e0235076. doi: 10.1371/journal.pone.0235076 (PMC7316246; doi:10.1371/journal.pone.0235076)
Supplement: S1 Table — List of all biological drug products considered in this analysis. (PDF) [file pone.0235076.s002.pdf]

Therapeutic Peptide Name	Brand Name	Therapeutic Category	Route of Administration[a]	Number of Excipients[b]
Abatacept	Orencia	Antirheumatic Agents and Immunosuppressive Agents	IV, SQ	3; 5
Abciximab	ReoPro	Anticoagulants	IV	4
Adalimumab	Humira	Antirheumatic Agents	SQ	9; 3
Aflibercept	Eylea	Antineoplastic Agents and Ophthalmics	intravitreal	4
Aflibercept	Zaltrap	Antineoplastic Agents and Ophthalmics	IV	4
Agalsidase beta	Fabrazyme	Enzyme Replacement Agents	IV	10
Albiglutide	Tanzeum	Drugs used in diabetes; alimentary tract and metabolism; blood glucose lowering drugs, excl. insulins.	SQ	4
Aldesleukin	Proleukin	Antineoplastic Agents	IV	5
Alemtuzumab	Campath	Antineoplastic Agents	IV	6
Alemtuzumab	Lemtrada	Antineoplastic Agents	IV	7
Alglucosidase alfa	Lumizyme	Enzyme Replacement Agents	IV	4
Alirocumab	Praluent	Alirocumab is indicated as an adjunct to diet and maximally tolerated statin therapy in adults who require additional LDL-cholesterol (LDL-C) lowering due to heterozygous familial hypercholesterolemia or clinical atherosclerotic cardiovascular disease.	SQ	4
Aliskiren	Tekturna	Renin inhibitor	PO	10
Aliskiren	Tekturna HCT	Renin inhibitor	PO	12
Alpha-1-proteinase inhibitor	Aralast	Serine Proteinase Inhibitors	IV	6
Alpha-1-proteinase inhibitor	Prolastin-C	For treatment of panacinar emphysema	IV	2
Alpha-1-proteinase inhibitor	Prolastin-C Liquid	For treatment of panacinar emphysema	IV	3
Alteplase	Activase	Thrombolytic Agents	IV	3
Anakinra	Kineret	Antirheumatic Agents	SQ	5
Anti-inhibitor coagulant complex	Feiba	Blood Coagulation Factors, Antihemophilic Agent	IV	2
Anti-thymocyte Globulin (Equine)	ATGAM	Antibody	IV	1
Antihemophilic Factor	Advate	Coagulants and Thrombotic agents	IV	8
Antihemophilic Factor	Alphanate	Coagulants and Thrombotic agents	IV	3
Antihemophilic Factor	Helixate FS	Coagulants and Thrombotic agents	IV	9
Antihemophilic Factor	Hemofil M	Coagulants and Thrombotic agents	IV	3

Antihemophilic Factor	Koate	Coagulants and Thrombotic agents	IV	4
Antihemophilic Factor	Kogenate FS	Coagulants and Thrombotic agents	IV	9
Antihemophilic Factor	Monoclate-P	Coagulants and Thrombotic agents	IV	5
Antihemophilic Factor	Xyntha	Coagulants and Thrombotic agents	IV	5
Antithrombin Alfa	Atryn	Thrombolytic Agents	IV	3
Antithrombin III human	Thrombate III	Thrombolytic Agents	IV	2
Asfotase Alfa	Strensiq	Enzymes Alimentary Tract and Metabolism	SQ	3
Asparaginase	Erwinaze	Antineoplastic Agents	IM, IV	2
Atezolizumab	Tecentriq	Antineoplastic Agents	IV	5
Basiliximab	Simulect	Immunosuppressive Agents	IV	6
Becaplermin	Regranex	Angiogenesis Inducing Agents	topical	9
Belatacept	Nulojix	Antirheumatic Agents and Immunosuppressive Agents	IV	3
Belimumab	Benlysta	Monoclonal antibodies	IV, SQ	4; 5
Beractant	Survanta	Beractant is indicated for Respiratory Distress Syndrome (RDS) in premature infants.	endotracheal	5
Bevacizumab	Avastin	Angiogenesis Inhibitors	IV	5
Bivalirudin	Angiomax	Antithrombins	IV	2
Blinatumomab	Blincyto	Antineoplastic Agents, Immunosuppressive Agents, Monoclonal antibodies, Antineoplastic and Immunomodulating Agents	IV	5
Botulinum Toxin Type A	Botox	Neuromuscular Blocking Agents, Anti-Wrinkle Agents and Antidystonic Agents	Intradermal, IM	2
Botulinum Toxin Type A	Dysport	Neuromuscular Blocking Agents, Anti-Wrinkle Agents and Antidystonic Agents	IM	2
Botulinum Toxin Type A	Xeomin	Neuromuscular Blocking Agents, Anti-Wrinkle Agents and Antidystonic Agents	IM	2
Botulinum Toxin Type B	Myobloc	Antidystonic Agents	IM	6
Brentuximab vedotin	Adcetris	Antineoplastic Agents	IV	4
C1 Esterase Inhibitor (Human)	Cinryze	Blood and Blood Forming Organs	IV	6
Canakinumab	Ilaris	Anti-Inflammatory Agents and Monoclonal antibodies	SQ	5; 5
Certolizumab pegol	Cimzia	TNF inhibitor	SQ	2; 3
Cetuximab	Erbitux	Antineoplastic Agents	IV	6
Chorionic Gonadotropin (Human)	Ovidrel	Hormones	SQ	6
Chorionic Gonadotropin (Human)	Pregnyl	Hormones	IM	4

Coagulation factor ix	Alprolix	Coagulants and Thrombotic Agents	IV	4
Coagulation factor VIIa	NovoSeven	Coagulants	IV	7
Coagulation Factor XIII A-Subunit (Recombinant)	Tretten	Antihemophilic Agents	IV	4
Collagenase	Santyl	Dermatologic agent	topical	1
Collagenase	Xiaflex	Dermatologic agent	intralesional	3
Conestat alfa	Ruconest	Blood and Blood Forming Organs	IV	3
Cosyntropin	Cortrosyn	Hormones and Diagnostic Agents	IM, IV	1
Daratumumab	Darzalex	Antineoplastic Agents	IV	5
Darbepoetin alfa	Aranesp	Anti-anemic Agents	IV, SQ	5
Denosumab	Xgeva	Bone Density Conservation Agents and Monoclonal antibodies	SQ	5
Denosumab	Prolia	Bone Density Conservation Agents and Monoclonal antibodies	SQ	5
Digoxin Immune Fab (Ovine)	DigiFab	Antidotes	IV	2
Dinutuximab	Unituxin	Antibody, Immunosuppressive agent, Antineoplastic agent	IV	5
Dornase alfa	Pulmozyme	Enzymes	respiratory (inhalation)	2
Dulaglutide	Trulicity	Hypoglycemic Agents; Drugs Used in Diabetes; Alimentary Tract and Metabolism; Blood Glucose Lowering Drugs, Excl. Insulins	SQ	5
Eculizumab	Soliris	For the treatment of patients with paroxysmal nocturnal hemoglobinuria (PNH) to reduce hemolysis.	IV	5
Elosulfase alfa	Vimizim	Enzymes; Alimentary Tract and Metabolism	IV	6
Elotuzumab	Empliciti	Antineoplastic Agents	IV	4
Enfuvirtide	FUZEON	HIV Fusion Inhibitors	SQ	4
Epoetin alfa	Epogen	Anti-anemic Agents	IV, SQ	4; 5
Epoetin zeta	Retacrit	Anti-anemic Agents	IV, SQ	14
Eptifibatide	Integrilin	Platelet Aggregation Inhibitors	IV	2
Etanercept	Enbrel	Immunosuppressive Agents	SQ	3; 3
Evolocumab	Repatha	Lipid Modifying Agents, Plain; Cardiovascular System	SQ	5
Exenatide	Byetta	Hypoglycemic Agents	SQ	5
Exenatide	Bydureon	Hypoglycemic Agents	SQ	8; 2
Factor IX Complex (Human)	AlphaNine SD	Antihemophilic agent	IV	4
Fibrinogen Concentrate (Human)	Riastap	Bleeding disorder	IV	7

Fibrinogen Concentrate (Human)	Evicel Fibrin Sealant (human)	Bleeding disorder	topical	6
Fibrinogen Concentrate (Human)	Tachosil	Bleeding disorder	topical	6
Filgrastim-sndz	Zarxio	Colony-Stimulating Factors and Thrombopoietic Agents	IV, SQ	5
Follitropin alpha	Gonal-F	Fertility Agents and Gonadotropins	SQ	5
Follitropin alpha	Gonal-f RFF	Fertility Agents and Gonadotropins	SQ	5
Follitropin beta	Follistim AQ	Fertility Agents	SQ	8
Galsulfase	Naglazyme	Enzyme Replacement Agents	IV	5
Gemtuzumab ozogamicin	Mylotarg	Antineoplastic agents and Immunotoxins	IV	5
Glatiramer acetate	Copaxone	Multiple Sclerosis Agents	SQ	1
Glucagon recombinant	GlucaGen	Hypoglycemic Agents	IM, IV, SQ	1
Glucagon recombinant	Glucagon	Hypoglycemic Agents	IM, IV	2
Glucarpidase	Voraxaze	Enzymes	IV	3
Golimumab	Simponi	Antipsoriatic Agents and Monoclonal antibodies and TNF inhibitor	SQ	5
Golimumab	Simponi Aria	Antipsoriatic Agents and Monoclonal antibodies and TNF inhibitor	IV	5
Hepatitis A Vaccine	Havrix	Vaccine	IM	2
Hepatitis B immune globulin	Hepagam B	Antiviral Agents	IM, IV	2
Hepatitis B immune globulin	Hyperhep B S/d	Antiviral Agents	IM	2
Hepatitis B immune globulin	Nabi-HB	Antiviral Agents	IM	3
Human clostridium tetani toxoid immune globulin	Hypertet S/d	It is indicated for prophylaxis against tetanus following injury in patients whose immunization is incomplete or uncertain (see below). It is also indicated, although evidence of effectiveness is limited, in the regimen of treatment of active cases of tetanus.	IM	2
Human rabies virus immune globulin	Hyperrab	It is indicated for individuals suspected of exposure to rabies, particularly severe exposure, with one exception: persons who have been previously immunized with HDCV Rabies Vaccine in a pre-exposure or postexposure treatment series should receive only vaccine.	IM, infiltration	2

Human rabies virus immune globulin	Imogam Rabies-HT	It is indicated for individuals suspected of exposure to rabies, particularly severe exposure, with one exception: persons who have been previously immunized with HDCV Rabies Vaccine in a pre-exposure or postexposure treatment series should receive only vaccine.	IM	3
Human Rho(D) immune globulin	Hyperrho S/d	NA	IM	2
Human Rho(D) immune globulin	Rhogam Ultra-filtered Plus	NA	IM	3
Human Rho(D) immune globulin	Rhophylac	NA	IM, IV	4
Human Rho(D) immune globulin	Winrho Sdf	NA	IM, IV	2
Human Serum Albumin	Albuminar	Serum substitutes	IV	4
Human Serum Albumin	Albutein	Serum substitutes	IV	4
Hyaluronidase	HYLENEX	Adjuvants, Anesthesia and Permeabilizing Agents	SQ	7
Ibritumomab	Zevalin	Antineoplastic Agents	IV	2
Idursulfase	Elaprase	Enzyme Replacement Agents	IV	4
Imiglucerase	Cerezyme	Enzyme Replacement Agents	IV	4
Immune Globulin Human	Bivigam	Immunologic Factors; Immunosuppressive Agents; Anti-Infective Agents	IV	4
Immune Globulin Human	Carimune Nanofiltered	Immunologic Factors; Immunosuppressive Agents; Anti-Infective Agents	IV	2
Immune Globulin Human	Flebogamma Dif	Immunologic Factors; Immunosuppressive Agents; Anti-Infective Agents	IV	2; 2
Immune Globulin Human	Gamastan	Immunologic Factors; Immunosuppressive Agents; Anti-Infective Agents	IM	2
Immune Globulin Human	Hyqvia	Immunologic Factors; Immunosuppressive Agents; Anti-Infective Agents	SQ	1
Infliximab	Remicade	Antirheumatic Agents, Anti-Inflammatory Agents, Non-Steroidal, Dermatologic Agents, Gastrointestinal Agents and Immunosuppressive Agents	IV	4
Insulin aspart	NovoLog	Hypoglycemic Agents and Antidiabetic Agents	IV, SQ	8
Insulin aspart	NovoLog Mix 70/30	Hypoglycemic Agents and Antidiabetic Agents	SQ	9
Insulin Degludec	Tresiba	Antidiabetic Agents	SQ	7
Insulin detemir	Levemir	Antidiabetic Agents	SQ	8

Insulin Glargine	Lantus	Hypoglycemic Agents	SQ	7
Insulin Glargine	Lantus Solostar	Hypoglycemic Agents	SQ	6
Insulin glulisine	Apidra	Antidiabetic Agents	SQ	5
Insulin Lispro	Humalog	Hypoglycemic Agents	IV, SQ	8; 8
Insulin, porcine	Vetsulin	Hypoglycemic Agents	SQ	5
Insulin,isophane	Novolin N	Hypoglycemic Agents and Antidiabetic Agents	SQ	9
Interferon alfa-2b	Intron A	Immunosuppressive Agents	IM, IV, SQ, Intralesional	4; 6
Interferon beta-1a	Avonex	Antineoplastic Agents	IM	5
Interferon beta-1a	Betaseron	Multiple Sclerosis Agents	SQ	2
Interferon beta-1a	Extavia	Multiple Sclerosis Agents	SQ	2
Interferon beta-1a	Rebif	Multiple Sclerosis Agents	SQ	4
Interferon gamma-1b	Actimmune	Immunosuppressive Agents	SQ	5
Intravenous Immunoglobulin	Flebogamma	IVIg	IV	2; 2
Intravenous Immunoglobulin	Gamunex	IVIg	IV, SQ	2
Ipilimumab	Yervoy	Antineoplastic Agents and Monoclonal antibodies	IV	6
Ixekizumab	Taltz	Antipsoriatic Agents and Monoclonal antibodies and TNF inhibitor	SQ	5
Laronidase	Aldurazyme	Enzyme Replacement Agents	IV	5
Leuprolide	Eligard	Antineoplastic Agents	SQ	2
Liraglutide	Saxenda	Antidiabetic Agents	SQ	4
Liraglutide	Victoza	Antidiabetic Agents	SQ	4
Menotropins	Menopur	Fertility Agents	SQ	3
Mepolizumab	Nucala	Antineoplastic and Immunomodulating Agents, Immunosuppressive Agents, Interleukin Inhibitors	SQ	5; 3
Methoxy polyethylene glycol-epoetin beta	Mircera	Anti-anemic Agents	IV	5
Metreleptin	Myalept	Lipodystrophy	SQ	4
Natalizumab	Tysabri	Immunosuppressive agents	IV	5
Necitumumab	Portrazza	Antineoplastic Agents	IV	7
Nesiritide	NATRECOR	For the intravenous treatment of patients with acutely decompensated congestive heart failure who have dyspnea at rest or with minimal activity.	IV	3
Nivolumab	Opdivo	Antineoplastic and Immunomodulating Agents	IV	8
Obiltoxaximab	Anthim	Investigated for use/treatment in anthrax exposure, bacterial infection, crohn's disease, and graft versus host disease.	IV	3
Obinutuzumab	Gazyva	Antineoplastic Agents	IV	5
Ocriplasmin	Jetrea	Ophthalmic	intravitreal	5

Ofatumumab	Arzerra	Antineoplastic and Immunomodulating Agents	IV	7
Omalizumab	Xolair	Anti-Allergic Agents	SQ	4; 5
Oxytocin	Pitocin	Oxytocics, Anti-tocolytic Agents and Labor Induction Agents	IV	3; 2
Palifermin	Kepivance	Anti-Mucositis Agents	IV	4
Palivizumab	Synagis	Antiviral Agents	IM	3
Pancrelipase	Viokace	Gastrointestinal Agents and Enzyme Replacement Agents	PO	6
Pancrelipase	Pertzye	Gastrointestinal Agents and Enzyme Replacement Agents	PO	8
Pancrelipase	Zenpep	Gastrointestinal Agents and Enzyme Replacement Agents	PO	13
Panitumumab	Vectibix	Antineoplastic Agents	IV	3
Pegaptanib	Macugen	Intended for the prevention of respiratory distress syndrome (RDS) in premature infants at high risk for RDS.	intravitreal	6
Pegaspargase	Oncaspar	Antineoplastic Agents	IM, IV	4
Pegfilgrastim	Neulasta	Immunosuppressive Agents	SQ	5
Peginterferon alfa-2a	Pegasys	Immunosuppressive Agents, Antineoplastic	SQ	6
Peginterferon alfa-2b	PEGIntron	Immunosuppressive Agents	SQ	4
Peginterferon beta-1a	Plegridy	Multiple Sclerosis Agents	SQ	4
Pegloticase	Pegloticase (Krystexxa)	Enzymes	IV	4
Pegvisomant	Somavert	Hormone Replacement Agents	SQ	4
Pembrolizumab	Keytruda	Antineoplastic and Immunomodulating Agents	IV	5; 4
Pertuzumab	Perjeta	Monoclonal antibodies	IV	5
Poractant alfa	Curosurf	Pulmonary Surfactants	endotracheal	2
Pramlintide	Symlin	Antidiabetic Agents	SQ	5
Protamine sulfate	Protamine Sulfate	Heparin Antagonists, Hematologic Agents	IV	3
Prothrombin complex concentrate	Kcentra	Antihemophilic Agents	IV	7
Ragweed Pollen Extract	Ragwitek	Anti-Allergic Agents	sublingual	3
Ramucirumab	Cyramza	Antineoplastic and Immunomodulating Agents	IV	6
Ranibizumab	Lucentis	Ophthalmic	intravitreal	5
Rasburicase	Elitek	Gout Suppressants	IV	3
Raxibacumab	Raxibacumab	Anti-Infective Agents and Monoclonal antibodies	IV	5
Reteplase	Retavase	Fibrinolytic Agents	IV	5
Rilonacept	Arcalyst	Immunosuppressive Agents	SQ	5
Rituximab	Rituxan	Antineoplastic Agents, Immunologic Factors and Antirheumatic Agents	IV	4
Romiplostim	Nplate	Colony-Stimulating Factors and Thrombopoietic Agents	SQ	5
Sacrosidase	Sucraid	Enzymes	PO	1

Salmon Calcitonin	Miacalcin	Used for the treatment of post-menopausal osteoporosis.	IM, SQ	5
Sargramostim	Leukine	Immunosuppressive Agents	IV, SQ	5; 6; 4; 3
Sebelipase alfa	Kanuma	Enzymes	IV	3
Secukinumab	Cosentyx	Antipsoriatic Agents and Monoclonal antibodies and TNF inhibitor	SQ	7
Serum albumin	Optison	Serum substitutes	IV	3
Serum albumin iodonated	Megatope	Diagnostic Agents	IV	2
Serum albumin iodonated	Volumex	Diagnostic Agents	IV	2
Siltuximab	Sylvant	Antineoplastic and Immunomodulating Agents, Immunosuppressive Agents	IV	4
Sipuleucel-T	Provenge	Antineoplastic and Immunomodulating Agents	IV	5
Susoctocog alfa	Obizur	Blood coagulation factors, Antihemorrhagics	IV	7
Teduglutide	Gattex	Treatment of short bowel syndrome (SBS), malabsorption associated with the removal of the intestine, in adults patients who are dependent on parenteral support.	SQ	4
Tenecteplase	TNKase	Fibrinolytic Agents	IV	3
Teriparatide	Forteo	Bone Density Conservation Agents	SQ	7
Tesamorelin	Egrifta	Tesamorelin acetate is a synthetic analogue of human hypothalamic Growth Hormone Releasing Factor (hGRF) indicated to induce and maintain a reduction of excess abdominal fat in HIV-infected patients with lipodystrophy.	SQ	1; 4
Thyrotropin Alfa	Thyrogen	Diagnostic Agents	IM	3
Tocilizumab	Actemra	Antirheumatic Agents	IV, SQ	4; 7
Trastuzumab	Herceptin	Antineoplastic Agents	IV	4
Tuberculin Purified Protein Derivative	Aplisol	Diagnostic Agents	intradermal	2
Urokinase	Kinlytic	Urokinase can be used for the treatment of pulmonary embolism, coronary artery thrombosis, IV catheter clearance, and venous and arterial blood clots.	IV	3
Ustekinumab	Stelara	Dermatologic agent, Immunosuppressive agent, antineoplastic agent	IV, SQ	5; 6
Vedolizumab	Entyvio	Immunosuppressive agent, Antineoplastic agent	IV	5
Velaglucerase alfa	Vpriv	Enzymes	IV	4

[a]IM = intramuscular; IV = intravenous; PO = oral; SQ = subcutaneous

[b]Numbers separated by a semicolon indicate number of excipients in each formulation of the respective medicine.
