# Supplementary material for: Biologic excipients: Importance of clinical awareness of inactive ingredients
Source: PLoS One. 2020 Jun 25;15(6):e0235076. doi: 10.1371/journal.pone.0235076 (PMC7316246; doi:10.1371/journal.pone.0235076)
Supplement: S2 Table — (PDF) [file pone.0235076.s003.pdf]

Ingredients					
**Acetic acid**	Acetate ion	Acetic acid	--	--	--
**Citric acid**	Anhydrous citric acid	Citric acid	Citric acid monohydrate	--	--
**Dextrose**	Dextrose	Dextrose monohydrate	--	--	--
**Histidine monohydrochloride**	Histidine monohydrochloride	Histidine monohydrochloride monohydrate	--	--	--
**Lactose**	Lactose	Lactose monohydrate	--	--	--
**Polyethylene glycol**	Polyethylene glycol	Polyethylene glycols	--	--	--
**Sodium acetate**	Sodium acetate	Sodium acetate anhydrous	Sodium acetate trihydrate	--	--
**Sodium citrate**	Sodium citrate	Trisodium citrate dihydrate	--	--	--
**Sodium phosphate dibasic**	Sodium phosphate dibasic	Sodium phosphate dibasic anhydrous	Sodium phosphate dibasic dihydrate	Sodium phosphate dibasic dodecahydrate	Sodium phosphate dibasic heptahydrate
**Sodium phosphate monobasic**	Sodium phosphate monobasic	Sodium phosphate monobasic dihydrate	Sodium phosphate monobasic monohydrate	--	--
**Trehalose**	Trehalose	Trehalose dihydrate	--	--	--
**Zinc**	Zinc	Zinc acetate	--	--	--
