# Supplementary material for: Biologic excipients: Importance of clinical awareness of inactive ingredients
Source: PLoS One. 2020 Jun 25;15(6):e0235076. doi: 10.1371/journal.pone.0235076 (PMC7316246; doi:10.1371/journal.pone.0235076)
Supplement: S3 Table — List of all ingredients that occur in biological formulations. Percentage occurrence refers to the fraction of all biological formulations analyzed that contain the ingredient. (PDF) [file pone.0235076.s004.pdf]

Ingredient	Number of formulation occurrences	Percentage occurrence in formulations
water	93	40.43
sodium chloride	88	38.26
polysorbate 80	66	28.70
sucrose	56	24.35
mannitol	48	20.87
sodium phosphate dibasic	47	20.43
histidine	40	17.39
sodium hydroxide	37	16.09
polysorbate 20	36	15.65
sodium phosphate monobasic	35	15.22
hydrochloric acid	31	13.48
sodium citrate	28	12.17
glycine	27	11.74
albumin (human)	24	10.43
citric acid	20	8.70
sodium acetate	18	7.83
histidine monohydrochloride	17	7.39
acetic acid	15	6.52
metacresol	15	6.52
sorbitol	12	5.22
arginine hydrochloride	11	4.78
phenol	11	4.78
zinc	11	4.78
glycerin	10	4.35
tromethamine	10	4.35
calcium chloride	9	3.91
methionine	9	3.91
trehalose	9	3.91
benzyl alcohol	7	3.04
edetate disodium	7	3.04
lactose	7	3.04
arginine	6	2.61
phosphoric acid	6	2.61
sodium phosphate	6	2.61
talc	5	2.17
alanine	4	1.74
cellulose microcrystalline	4	1.74
poloxamer 188	4	1.74
polyethylene glycol	4	1.74
potassium chloride	4	1.74
silicon dioxide	4	1.74
chloride ion	3	1.30
glutamic acid	3	1.30
hypromelloses	3	1.30
magnesium stearate	3	1.30
maltose	3	1.30
poly(dl-lactic-co-glycolic acid)	3	1.30
polyethylene glycol 3350	3	1.30
potassium phosphate monobasic	3	1.30
sodium bicarbonate	3	1.30
titanium dioxide	3	1.30
tri-n-butyl phosphate	3	1.30
tromethamine hydrochloride	3	1.30
calcium	2	0.87

carboxymethylcellulose sodium	2	0.87
chlorobutanol	2	0.87
copper	2	0.87
croscarmellose sodium	2	0.87
crospovidone	2	0.87
dextrose	2	0.87
ferric oxide red	2	0.87
heparin	2	0.87
lysine hydrochloride	2	0.87
methylparaben	2	0.87
pentetic acid (DTPA)	2	0.87
propylene glycol	2	0.87
protamine sulfate	2	0.87
sodium acetyltryptophanate	2	0.87
sodium caprylate	2	0.87
sodium carbonate	2	0.87
sodium polymetaphosphate	2	0.87
sodium succinate hexahydrate	2	0.87
threonine	2	0.87
acetyltryptophan	1	0.43
aluminum hydroxide	1	0.43
ammonium acetate	1	0.43
antithrombin III human	1	0.43
caprylic acid	1	0.43
carrageenan	1	0.43
cellulose acetate phthalate	1	0.43
colfosceril palmitate	1	0.43
dextran 40	1	0.43
diethyl phthalate	1	0.43
diglycine	1	0.43
disodium hydrogen citrate	1	0.43
equine collagen	1	0.43
ferrosoferric oxide	1	0.43
glutathione	1	0.43
human immunoglobulin A	1	0.43
hydrogenated castor oil	1	0.43
hypromellose phthalate	1	0.43
isoleucine	1	0.43
lactic acid	1	0.43
leucine	1	0.43
marine collagen soluble	1	0.43
methyl pyrrolidone	1	0.43
nitrogen	1	0.43
palmitic acid	1	0.43
petrolatum	1	0.43
phenylalanine	1	0.43
polyvinylpyrrolidone	1	0.43
potassium phosphate dibasic	1	0.43
povidone	1	0.43
proline	1	0.43
propylparaben	1	0.43
riboflavin	1	0.43
sodium lactate	1	0.43
sodium lauryl sulfate	1	0.43
sodium starch glycolate	1	0.43
sodium sulfate anhydrous	1	0.43

starch wheat	1	0.43
stearic acid	1	0.43
succinic acid	1	0.43
sulfuric acid	1	0.43
tranexamic acid	1	0.43
triethyl citrate	1	0.43
tripalmitin	1	0.43
ursodiol	1	0.43
valine	1	0.43
zinc chloride	1	0.43
